# Proximal-type epithelioid sarcoma of the perineum: A case report and literature review

**DOI:** 10.3389/fonc.2023.1057466

**Published:** 2023-03-06

**Authors:** Shijun Xia, Wenjiang Wu, Lijuan Ma

**Affiliations:** ^1^ Shenzhen Hospital of Guangzhou University of Chinese Medicine, Shenzhen, China; ^2^ Shenzhen Traditional Chinese Medicine Anorectal Hospital, Shenzhen, China

**Keywords:** proximal-type, epithelioid sarcoma, perineum, diagnosis, resect

## Abstract

Proximal-type epithelioid sarcoma of the perineum is a rare soft-tissue malignancy, and only 55 cases have been reported in the English literature to date. This tumor has an indetectable early symptom and frequent recurrences. Here, we present the case of a 31-year-old man with proximal-type epithelioid sarcoma of the perineum who underwent wide excision. Further, we reviewed the current literature regarding differential diagnosis and management of this disease.

## 1 Introduction

Franz Enzinger described 62 cases of a peculiar sarcoma, initially named epithelioid sarcoma (ES) in 1970, which was frequently confused with chronic inflammation, squamous cell carcinoma, and necrotizing granuloma (1). ES is a rare, aggressive soft-tissue malignancy characterized by nodular aggregates of epithelioid cells.

This tumor accounts for less than 1% of adult soft-tissue sarcomas and 4%–8% of pediatric sarcomas (2, 3). It is most prevalent among young adults, with higher prevalence among men than among women. ES can be divided into classic and proximal-type variants.

ES of the perineum was first described in 1997 (4). PubMed and Embase were searched, and relevant literature published until January 30, 2022, were retrieved by LJ-Ma using the following search terms: “epithelioid sarcoma” OR “epithelioid sarcomas” AND “perineum.” A total of 55 cases were reported between 1997 and 2022 (**Table 1**). In this article, we report the 56th case of proximal-type ES of the perineum.

**Table 1:** Reported cases of ES of the perineum.

Case	Study	Age	Sex	Country	Therapy	Recurrence	Follow-up
1	Sonobe et al,1997 (4)	60	M	Japan	E, C, R	YES	23months, DOD
2	Grobmyer et al,2001 (5)	28	M	USA	E, R	YES	112months, NED
3	Grobmyer et al,2001 (5)	34	F	USA	E, R	YES	81months, NED
4	Grobmyer et al,2001 (5)	37	M	USA	E, R	YES	51months, DOD
5	Grobmyer et al,2001 (5)	41	M	USA	E, R	NA	9months, NED
6	Hasegawa T et al, 2001 (6)	52	M	Japan	E	NA	36months, NED
7	González et al, 2001 (7)	84	F	Spain	E, R	YES	NA
8-10	Tateishi U et al, 2002 (8)	NA	NA	Japan	E	NA	NA
11	Humble SD et al, 2003 (9)	52	M	USA	E, R	NA	24months, NED
12	Chaudhuri A et al, 2003 (10)	54	M	UK	E, C, R	NA	NA
13	Masunaga A et al, 2004 (11)	36	M	Japan	E	YES	4months, DOD
14	Ikeda K et al, 2005 (12)	36	M	Japan	E	YES	3months, DOD
15	Zevallos et al, 2005 (13)	51	M	Saudi Arabia	E, R	NA	NA
16	Zevallos et al, 2005 (13)	43	M	Saudi Arabia	E, C, R	YES	NA
17	Dainese E et al, 2005 (14)	34	f	Italy	NA	NA	NA
18	Miyake M et al, 2006 (15)	31	M	Japan	E, C, R	NA	14months, NED
19	Hikosaka A et al, 2006 (16)	46	M	Japan	E, C	YES	39months, NED
20	Wei QZ et al, 2006 (17)	51	M	China	E	NA	NA
21	Rekhi B et al, 2007 (18)	47	M	India	E, C, R	YES	8months, NED
22-23	Wang CN et al, 2009 (19)	NA	NA	China	E	NA	NA
24	Flucke U et al, 2010 (20)	22	F	Germany	E	NA	NA
25	Tholpady A et al, 2010 (21)	17	F	USA	E	NA	12months, NED
26	Ong AC et al, 2012 (22)	51	F	Singapore	E	NA	8months, NED
27	Kim HJ et al, 2012 (23)	41	F	Korea	E, R	NA	10months, NED
28	Murashima T et al, 2013 (24)	24	M	Japan	E	NA	12months, NED
29-30	Cossu A et al, 2013 (25)	NA	NA	NA	NA	NA	NA
31	Patrizi L et al, 2013 (26)	63	F	Italy	E, R	YES	14months, NED
32-35	Li Li et al, 2014 (27)	NA	NA	China	E	NA	NA
36	Rodrigues AI et al, 2015 (28)	55	F	Portugal	NA	YES	16days, DOD
37	Folpe AL et al, 2015 (29)	55	F	USA	E	YES	18months, DOD
38	Folpe AL et al, 2015 (29)	35	F	USA	E	YES	24months, DOD
39	Folpe AL et al, 2015 (29)	59	F	USA	E	NA	54months, NED
40	Folpe AL et al, 2015 (29)	34	F	USA	E, C, R	YES	19months
41	Folpe AL et al, 2015 (29)	46	F	USA	E	NA	5months, NED
42	Folpe AL et al, 2015 (29)	43	F	USA	E	NA	NA
43	Rekhi B et al, 2016 (30)	45	M	India	E	NA	NA
44	Sundaram A et al, 2018 (31)	36	F	India	R	YES	NA
45	Yue Y et al, 2018 (32)	41	F	China	E	NA	9months, NED
46	Pol JN et al, 2019 (33)	32	M	India	E	NA	NA
47	Noh JJ et al, 2021 (34)	29	F	Korea	E, R	NA	24months, NED
48	Noh JJ et al, 2021 (34)	35	F	Korea	E, C, R	YES	NA
49	Noh JJ et al, 2021 (34)	24	F	Korea	E, R	YES	NA
50	Chung H et al, 2021 (35)	24	F	Korea	C, R	YES	19months, DOD
51	Orita Y et al, 2022 (36)	36	F	Japan	E, C	NA	60months, NED
52	Dash B et al, 2022 (37)	45	F	India	E	YES	72months, NED
53	Dash B et al, 2022 (37)	27	F	India	E	NA	24months, NED
54	Dash B et al, 2022 (37)	37	F	India	E	NA	12months, NED
55	Yahiro S et al, 2022 (38)	24	F	Japan	E, R	YES	6months, DOD

M, male; F, female; E, excision; C, chemotherapy; R, radiotherapy; NA, information not available; DOD, dead of disease; NED, no evidence disease.

## 2 Case description

**Figure 1:**
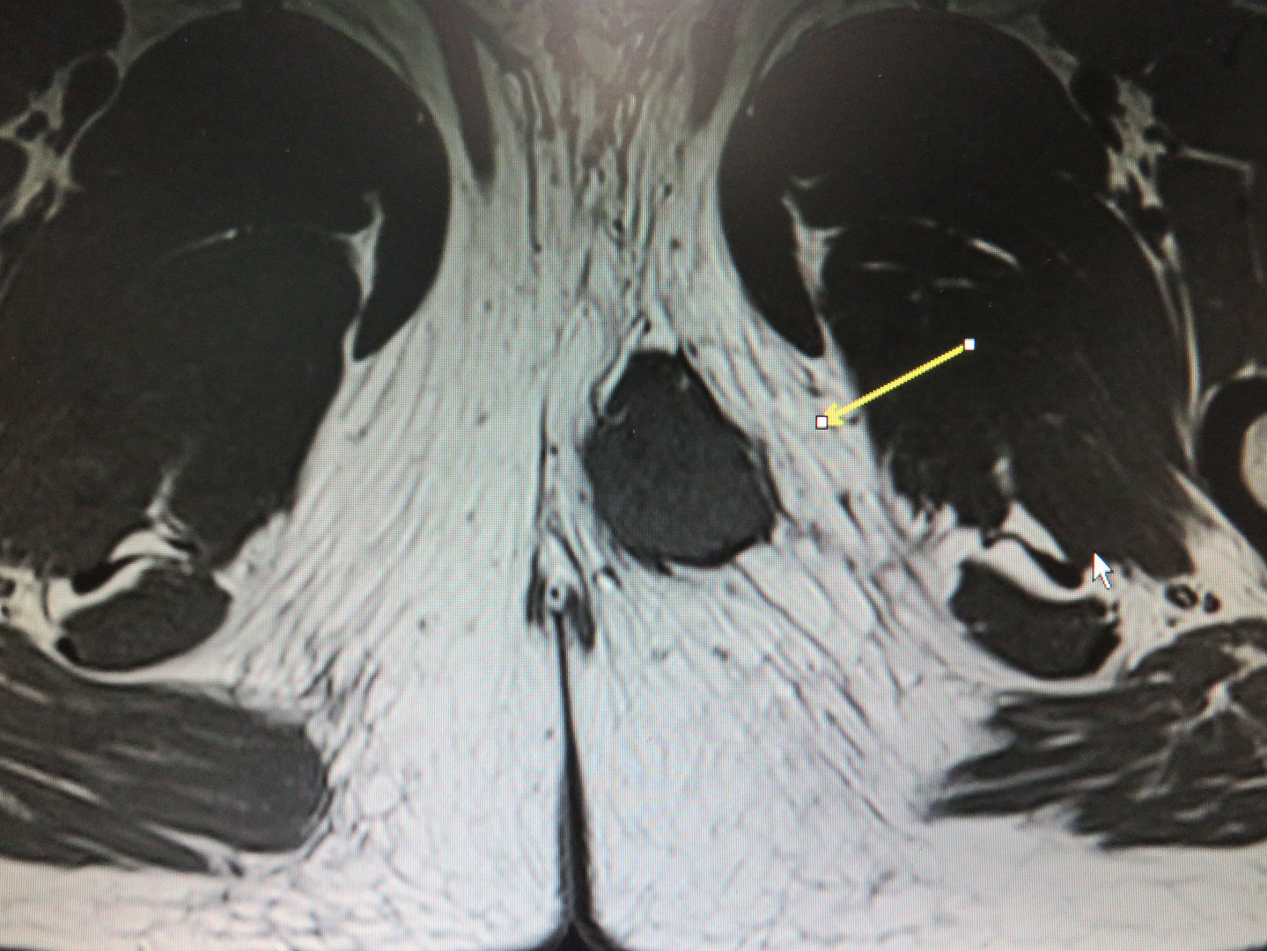
MRI revealed a perineal tumor. The arrow points out the location of the lump.

**Figure 2:**
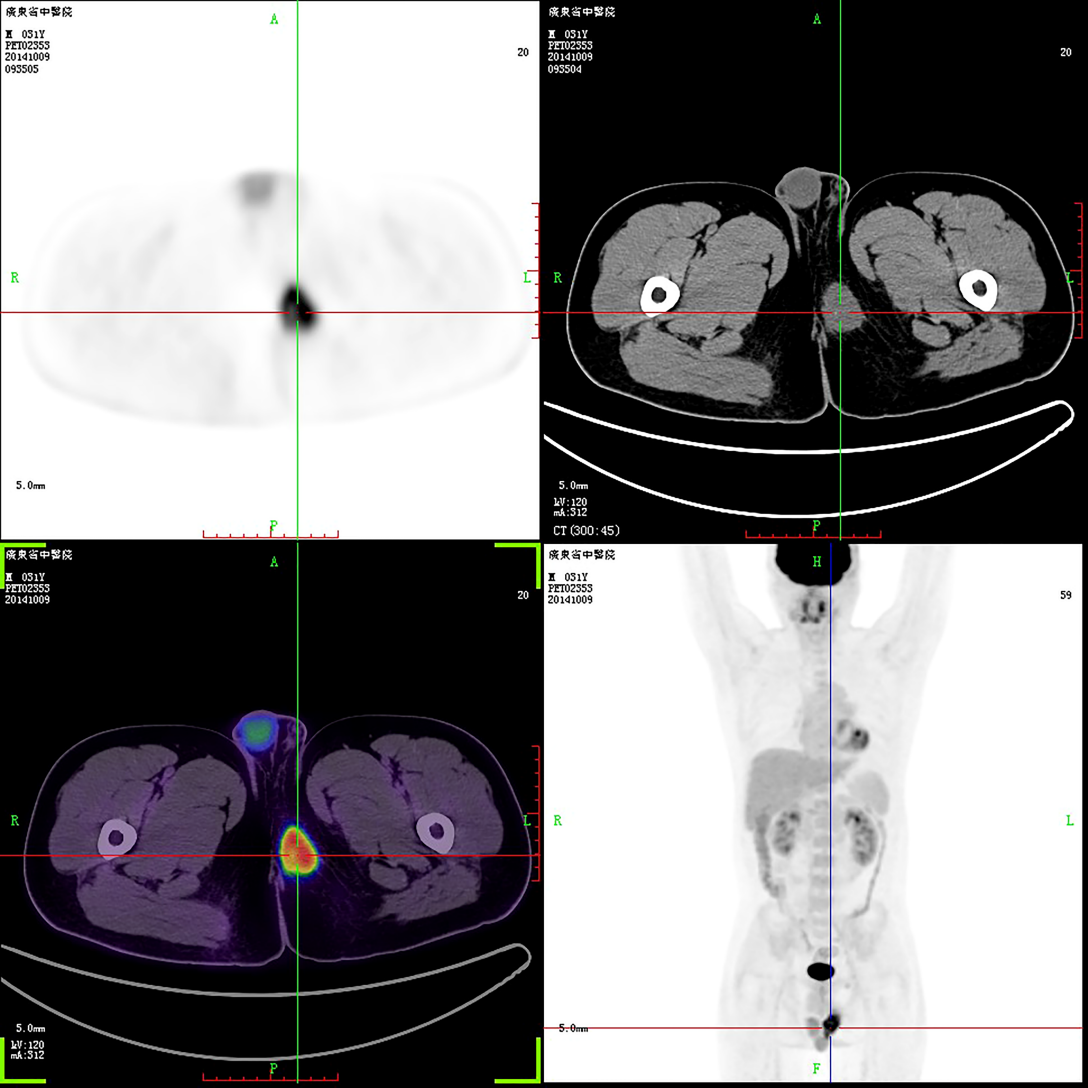
PET-CT revealed a perineal tumor. The purpose of uppercase letters is to mark the position.

A 31-year-old man was admitted to a local hospital with a painless lump in the right perineum region, for which he received surgical incision and drainage. Five months after surgery (September 2019), he visited our hospital for further treatment because of poor healing of the incision accompanied by pain and swelling. Magnetic resonance imaging (MRI) revealed a well-circumscribed solid and homogeneous lump in the right perineum (**Figure 1**). Positron emission tomography-computed tomography (PET-CT) for initial staging showed a heterogeneous hypermetabolic mass in the right perineum and a fistula in the lump (**Figure 2**). However, no bone or skin involvement or lung or liver metastases were noted. His medical history was unremarkable. The patient underwent complete resection with negative margins of the tumor through a perianal incision and avoided damaging the rectum and anal sphincter, followed by 25 courses of adjuvant radiotherapy at a dose of 50 Gray targeting the right perineum. Specifically, the mass was excised with all margins free of tumor, and there was about a 1-cm distance between the tumor lesion and the excised margin in all directions. At the 18-month follow-up, the patient was still alive and free of disease.

**Figure 3:**
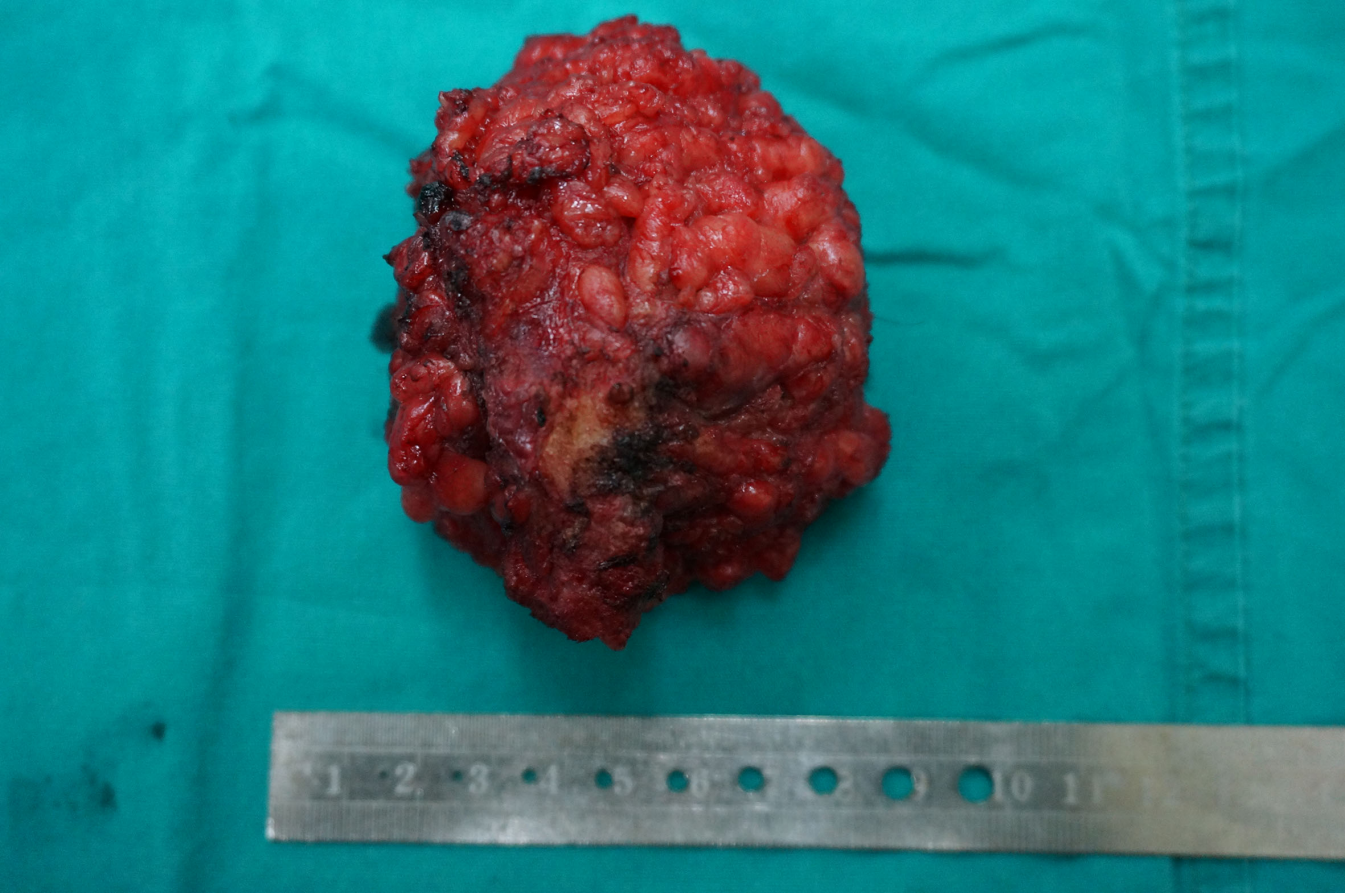
Macroscopic image of the resected specimen.

**Figure 4:**
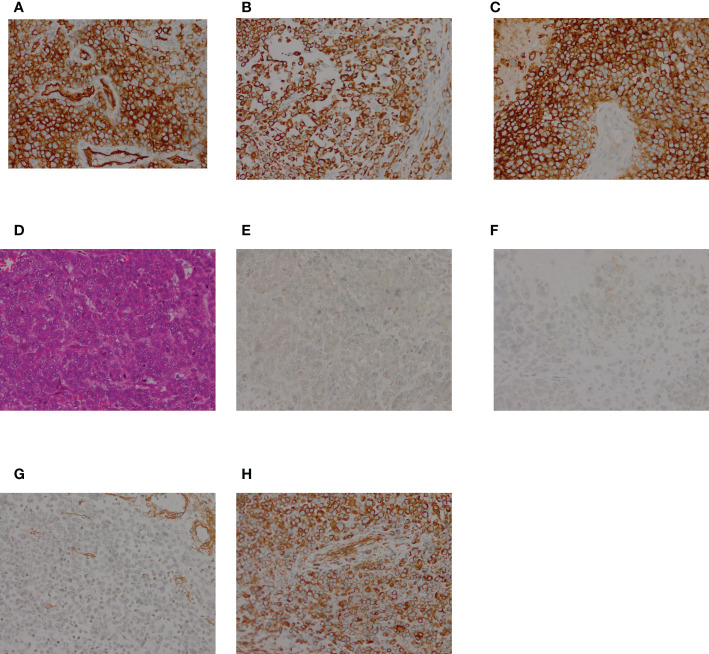
HE and immunohistochemical staining of the resected specimen. **(A)** HE staining showing polygonal tumor cells; (B–I) Immunohistochemical staining. The tumor cells were positive for CK **(B)**, EMA **(C)**, Vimentin **(D)**, SMA **(G)**, and CD34 **(H)**, and negative forMyoD1 **(E)**, and Myogenin **(F)**.

Macroscopically, the resected oval-shaped specimen measured 8 × 10 × 5 cm and was enclosed by a rim of fatty tissue (**Figure 3**). The incisal surface showed dispersive areas of necrosis and hemorrhage. Immunohistochemical staining indicated that the tumor cells were CK (portion +), EMA (+), Vimentin (+), CK7 (–), CK20 (–), HMB45 (–), MyoD1 (–), Myogenin (–), Desmin (–), SMA (+), Actin (–), CD34 (+), S-100 (–), Calretinin (–), CD31 (–), and Bcl-2 (–) (**Figure 4**).

## 3 Discussion

Proximal-type ES is an uncommon but aggressive soft-tissue malignancy with a poor prognosis. However, deficient information is available in the literature regarding the clinical aspects and management of proximal-type ES of the perineum. **Table 1** summarizes cases of proximal-type ES of the perineum reported since 1997. Based on the available data from these reports, the average age of the 55 patients was 40.8 years. Among them, 18 patients (40.9%) were men, 26 were women (59.1%), and the sex was not specified for the remaining 11. In subgroups stratified by ethnicity, Asians had a higher incidence than other ethnic groups. Our patient is the 66th reported case of proximal-type ES of the perineum.

Seeking immediate treatment is uncommon in these patients because the tumor is often painless. Therefore, a proximal-type ES located deep in the perineum may become quite large at the initial diagnosis. Plain radiography, MRI, CT, and PET can help estimate the volume of the soft tissue mass and determine its metastaticity. Moreover, histological diagnosis of the soft tissue mass is essential.

Unlike most soft-tissue sarcomas that are classified according to the presumptive tissue of origin, such as liposarcoma, synovial sarcoma, or leiomyosarcoma, the histogenesis of ES remains unclear. Immunohistochemical staining, however, may help identify the presumptive tissue of origin in patients with ES. ES is typically positive for vimentin, CK, and EMA.

Surgical excision with negative gross margins is the mainstay of therapy for patients with primary sarcoma. Generally, there is no evidence of distant metastasis at the initial diagnosis. For localized disease, adjuvant radiotherapy after wide surgical excision is necessary to reduce the risk of local recurrence. In a retrospective review of 1,170 patients with newly diagnosed soft tissue sarcoma during a 7.5-year period, the incidence of distant metastases at diagnosis was 10%, 83% of which were in the lungs (39). Hematogenous metastasis is the most common spread in patients with soft tissue sarcoma. Compared to the classical type, proximal-type ES is a more dreaded type with a poorer prognosis and trend of distant metastasis (40). Therefore, the 5-year overall survival rate of proximal- and classical types are 57% and 77%, respectively. Treatment options for proximal-type ES with distant metastasis usually involve surgical treatment, adjuvant radiotherapy, and/or adjuvant chemotherapy. For patients with local recurrence or metastatic disease, re-excision may be warranted.

Owing to the rarity of such tumors of the perineum, published treatment recommendations are unavailable. At present, total surgical resection combined with postoperative chemotherapy and radiotherapy is the major treatment modality for primary proximal-type ES. However, EZH2 inhibitors and immunotherapy may represent potential treatments for such tumors (41). New features of proximal-type ES and differences in responses to various treatments must be further explored to accumulate high-quality evidence for treatment suggestions.

## Data availability statement

The original contributions presented in the study are included in the article/supplementary material. Further inquiries can be directed to the corresponding author.

## Ethics statement

Ethical review and approval was not required for the study on human participants in accordance with the local legislation and institutional requirements. The patients/participants provided their written informed consent to participate in this study.

## Author contributions

SX: analysis and interpretation of data, drafting the article or revising it critically for important intellectual content; WW: final approval of the version to be published; LM: acquisition of data. All authors contributed to the article and approved the submitted version.
